# Amino Acid Repeats Cause Extraordinary Coding Sequence Variation in the Social Amoeba *Dictyostelium discoideum*

**DOI:** 10.1371/journal.pone.0046150

**Published:** 2012-09-28

**Authors:** Clea Scala, Xiangjun Tian, Natasha J. Mehdiabadi, Margaret H. Smith, Gerda Saxer, Katie Stephens, Prince Buzombo, Joan E. Strassmann, David C. Queller

**Affiliations:** 1 Department of Ecology and Evolutionary Biology, Rice University, Houston, Texas, United States of America; 2 Department of Biology, Washington University in St. Louis, St. Louis, Missouri, United States of America; 3 Department of Biochemistry and Cell Biology, Rice University, Houston, Texas, United States of America; University Of Montana - Missoula, United States of America

## Abstract

Protein sequences are normally the most conserved elements of genomes owing to purifying selection to maintain their functions. We document an extraordinary amount of within-species protein sequence variation in the model eukaryote *Dictyostelium discoideum* stemming from triplet DNA repeats coding for long strings of single amino acids. *D. discoideum* has a very large number of such strings, many of which are polyglutamine repeats, the same sequence that causes various human neurological disorders in humans, like Huntington’s disease. We show here that *D. discoideum* coding repeat loci are highly variable among individuals, making *D. discoideum* a candidate for the most variable proteome. The coding repeat loci are not significantly less variable than similar non-coding triplet repeats. This pattern is consistent with these amino-acid repeats being largely non-functional sequences evolving primarily by mutation and drift.

## Introduction

One of the strongest patterns in molecular evolution is that DNA sites affecting amino acid sequences are relatively invariant [1], so much so that invariance between species is used to help identify coding sequences [2]. The same pattern holds within species [3], except for rare loci with high amino acid variation maintained by balancing selection. The genome of the social amoeba *Dictyostelium discoideum* is unusual in having thousands of triplet-repeat microsatellites in genes, coding for long runs of a single amino acid [4]. Here we show that such microsatellites are extremely variable in repeat number, leading to highly variable genomic coding sequence. This raises evolutionary questions about the origin and maintenance of such extensive variation, and physiological questions about how *D. discoideum* protects itself against amino acid repeats that cause disease in humans.

Microsatellites are repetitive DNA sequences with unit motifs of 1 to 6 base pairs [5], [6]. The repetitive structure favors misaligned annealing during replication, and consequent changes in the number of repeats (slippage mutations) [7], [8]. The rate of slippage mutation is high, 10^−2^–10^−5^, leading to high variation which makes microsatellites good genetic markers [9]. Most studies have analyzed microsatellites in non-coding DNA, and find high polymorphism levels, as expected if natural selection is weaker in these regions [10], [11]. However, coding regions also contain microsatellites, particularly those with triplet motifs which can change in repeat number without causing reading frame shifts that would destroy gene function [12]. These too can show repeat number polymorphisms [9], and in some cases large repeat numbers cause pathology, such as Huntington’s disease [13]. The density of microsatellites and amino acid repeats are strongly influenced by the nucleotide composition [14], [15], which might suggest random generation by mutation and neutrality [16]. However, other recent research supports the hypothesis that these loci are naturally selected [17]–[20].

In *D. discoideum* repeats are occur more commonly in non-coding sequences than coding sequences, but not remarkably so (every 392 bp versus every 724) [4], suggesting that purifying selection to get rid of coding repeats is not particularly strong. However, coding repeats are dominated by a few amino acids, especially asparagine and glutamine but also threonine and serine, suggesting that repeats of other amino acids are often eliminated by selection [4]. The four amino acids just mentioned are all polar and non-hydrophobic, and may therefore loop outside of the protein and not disrupt its internal structure. Moreover, these repeats tend to occur in genes with low expression levels and high rates of change at synonymous sites, suggesting that they may not be strongly selected [21]. However, the opposite conclusion might be drawn from the fact that long repeats of Q and N are enriched in GO categories of protein kinases, lipid kinases, transcription factors, RNA helicases and messenger RNA and binding proteins such as spliceosome components [22]. Clearly, the question of functionality of these repeats is unresolved.

Low variability within a species is another indicator of the intensity of purifying selection. Some variability of coding-region microsatellites has been shown in humans [23] and in *Drosophila* [24], so we sought to determine whether the large number, and long length, of microsatellites in the coding DNA of *D. discoideum* leads to extraordinary sequence diversity or whether purifying selection maintains low diversity. We report variation data from three sets of microsatellite loci, with length scored on an automated sequencer after PCR amplification of the microsatellite regions. These coding microsatellites are as variable as non-coding microsatellites suggesting that they are not under stronger stabilizing selection.

## Results

### Triplet-nucleotide Microsatellites are Abundant in Coding Sequences of *D. discoideum*

**Figure 1:**
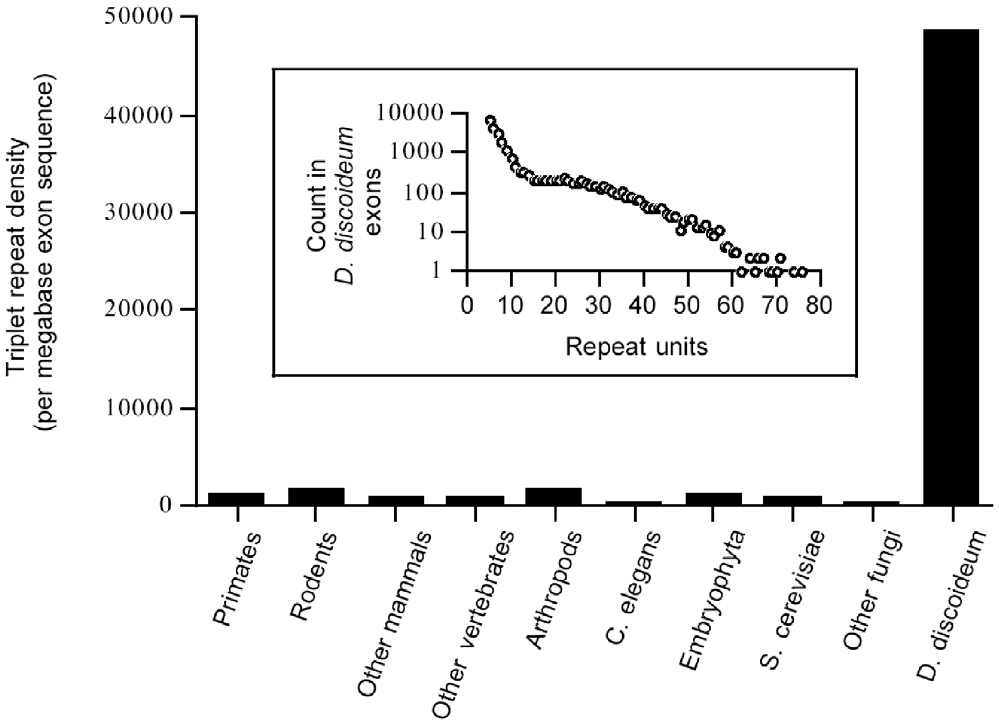
Triplet repeat density in *D. discoideum* exons. The inset shows the frequency distribution of triplet-repeat loci in exons of *D. discoideum*, grouped by the number of uninterrupted repeat units. The main figure histogram compares density of such exon repeats with other taxa, which were scored for triplet repeat of length 4 or higher in exons [39]. The *D. discoideum* data include all loci with 5 or more repeats and is therefore slightly conservative.

Microsatellites are extremely common in the genome of the social amoeba *Dictyostelium discoideum*, making up over 10% of the overall sequence [4]. Even more strikingly, triplet-repeat microsatellites are very common in the coding regions. Where triplet repeats typically make up roughly 0.1% of the coding sequence of various plants, animals, and fungi, the percentage in *D. discoideum* is about 50 times higher (Figure 1). The lengths of repeats are also exceptional, with 3064 perfect triplet-repeat microsatellites equaling or exceeding 20 repeats. The most common repeat motifs are AAT and CAA, leading to over 2966 tracts of 20 or more tandem iterations of the amino acids asparagine or glutamine.

### Expected Heterozygosity of Microsatellites Loci is Very High within Populations

Our attention was first directed to the problem by eight triplet-repeat loci (Table S1, last 8 loci) we were using as putative neutral markers for a population survey of diversity in 316 wild-collected haploid clones (Table S2), which we later determined occurred inside predicted coding regions. We call this the *clone-rich sample*. To explore more loci, we developed a *locus-rich sample*, choosing 49 microsatellites, with 27 to 40 repeats in the genome sequence, from the exons of 49 predicted genes widely distributed across all six chromosomes (Table S3). These were genotyped for 12 clones from 12 states in USA and also for 12 clones from a small geographic range in Virginia (Table S4). Finally, our *multiple-repeat sample* focused on three additional genes, with two goals in mind. First, these genes were chosen as representatives of the large fraction (18%) of *D. discoideum* genes with two or more amino acid repeats [4], so that we could assay the upper end of genic variability. Second, we chose loci that were not just predicted genes, but confirmed genes whose phenotypic effects have been studied. All three have effects during the starvation induced aggregation of cells to form a multicellular fruiting body: *dimA* is a transcription factor that regulates cell differentiation; *yakA* is a kinase involved in cell aggregation; and *atg1* is another kinase involved in recycling non-essential cellular components during starvation (Table S1). We genotyped 115 clones (a subset of those in clone-rich sample, see Table S2) at two triplet-repeat sequences in *atg1* and three each in *dimA* and *yakA*. These are not the only microsatellite repeats in these genes (Table S5), so our estimates of total diversity at these genes will be conservative.

**Figure 2:**
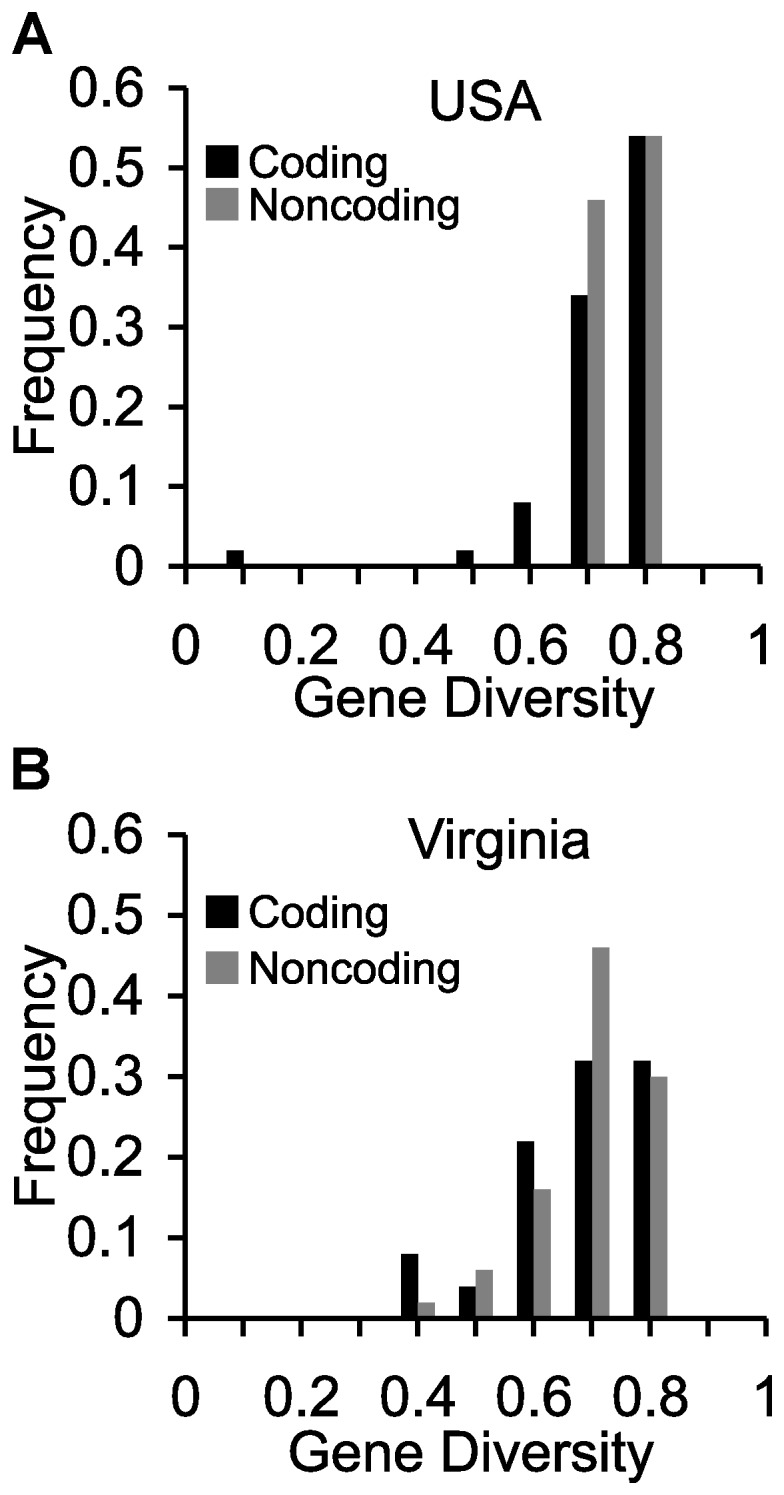
Gene diversity (expected heterozygosity) of the 50 coding triplet-repeat loci and 50 matched non-coding triplet repeat loci in locus-rich sample. (**a**) 12 clones isolated from different states of USA (**b**) 12 clones sampled over a 50 m transect in Virginia.

**Figure 3:**
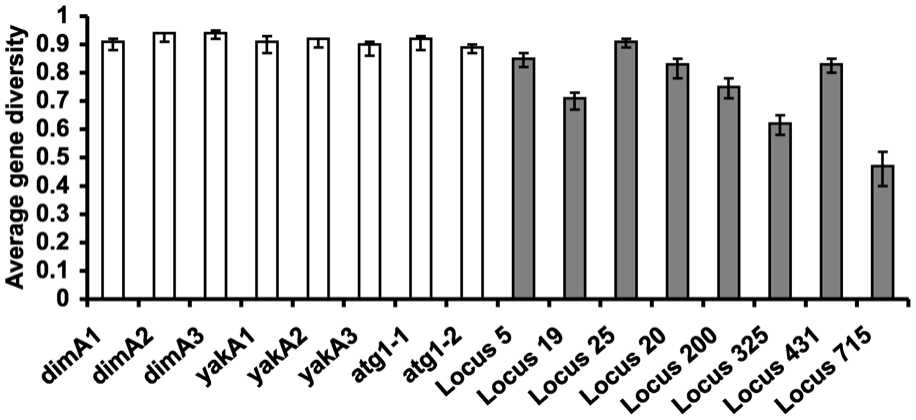
Gene diversity (expected heterozygosity) of microsatellites in the multiple-repeat and clone-rich samples. White bars show 8 microsatellites genotyped in the multiple-repeat sample, from the known genes *dimA*, *yakA* and *atg1*. Grey bars show the 8 microsatellite loci from the clone-rich sample of 316 clones. Error bars are 95% confidence intervals, estimated using 1000 bootstrap samples of individuals with replacement.

**Figure 4:**
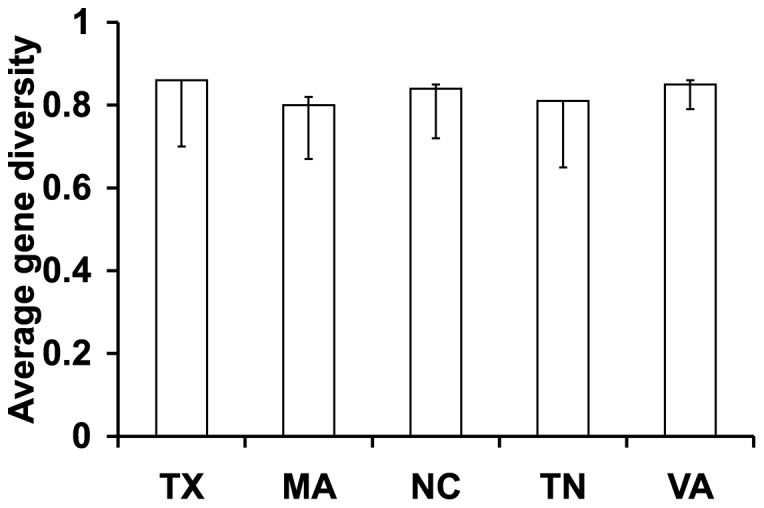
Average Gene diversity (expected heterozygosity) of the 8 triplet microsatellite loci in the multiple-repeat sample, divided by subpopulation. The error bars mark the bootstrap 95% confidence intervals, estimated using 1000 bootstrap samples of individuals with replacement.

Gene diversity, equivalent to expected heterozygosity or the probability that two randomly chosen alleles are different, is very high in all of these samples (Figs 2, 3). This is not explained by differentiation of isolated subpopulations because gene diversity values remain very high within subpopulations (Figure 4), including even the Virginia population sampled over a 50 m transect (Figure 2b). As expected from the fact that that longer microsatellites tend to undergo more slippage mutations [25], [26], genes with higher average numbers of repeats had greater diversity (linear regression for the 12 USA clones, y = 0.0093x+0.5435 *p* = 0.00003).

For the 50 coding loci of the repeat-rich sample, we had a sample of 50 non-coding loci matched for genome location, repeat motif, and repeat number. These non-coding loci did not have significantly different gene diversity than coding loci for either the USA sample (Fig. 2a, coding = 0.780, coding = 0.804, paired t test p = 0.14) or for the Virginia sample (Fig. 2b, coding = 0.713, coding = 0.730, paired t test p = 0.13).

### Multiple-repeat Samples Show High Amino Acid Diversity

**Figure 5:**
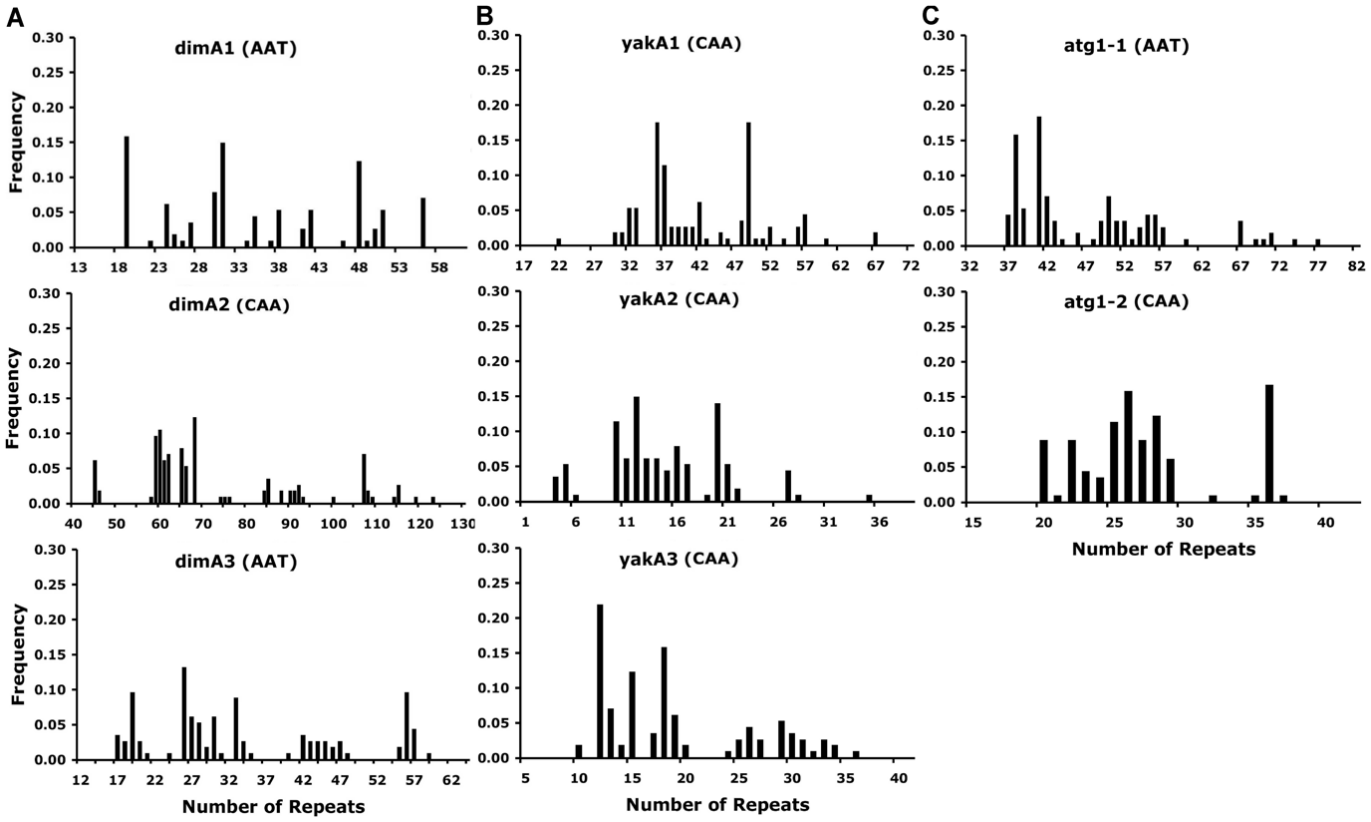
The frequency distribution of genotypes of eight coding triplet repeats from the gene *dimA, yakA,* and *atg1*. These were genotyped from a sample of 115 clones isolated from various North American locations.

**Figure 6:**
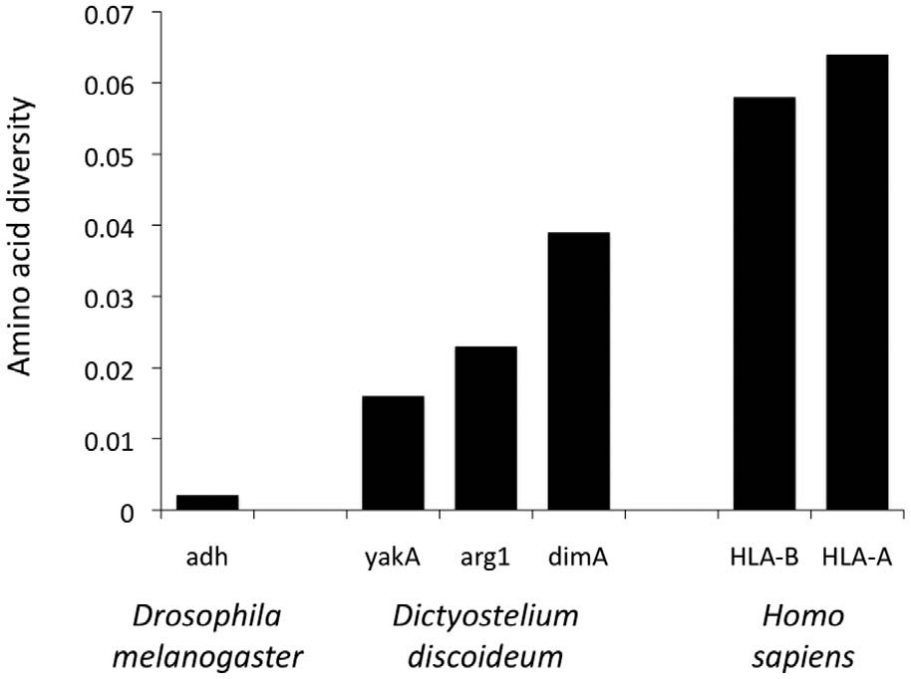
Amino acid diversity of the genes *yakA*, *arg1* and *dimA*. The average amino acid diversity (or the average heterozygosity per amino acid position) was calculated for *D. discoideum* for *yakA*, *arg1* and *dimA* loci using only variation in numbers of amino acids in the assayed repeats. The value of Drosophila *Adh* locus is from Kreitman [40] and Hartl [41], and values of Human *HLA-A* and *HLA-B* loci are from Hedrick [27], both based on substitutions.

The three genes where we assayed multiple repeats provide some indication of how variable entire genes can be. Figure 5 shows the extensive diversity in more detail for the the principal triplet-repeat regions in the *dimA, yakA* and *atg1* genes. To compare the resulting amino acid variation with other genes, we adapted Hedrick’s measure of amino acid expected heterozygosity [27], which we call amino acid diversity because it is analogous to the familiar measure of nucleotide diversity [28]. It measures the probability, averaged across all amino acid positions in a gene, that two amino acids drawn from different individuals will be different. We adapt this measure to include length differences (indels) by also scoring an amino acid position in an alignment as different when one individual possesses an amino acid at that position and the other does not. Figure 6 shows amino acid diversities for our three genes, underestimated somewhat because we used only the length differences at the assayed microsatellites, and considered all other sites identical. Also shown are the substitution-based diversities of the hyper-diverse human *HLA-A* and *HLA-B* loci and, for a more typical well studied gene, the Drosophila melanogaster *Adh* locus. The diversities of our three loci are not very much lower than the highly variable human *HLA* loci thought to be under strong balancing selection, and are many times higher than that of the *D. melanogaster Adh* locus.

In the *D. discoideum* genome there are 1423 coding sequences with at least one perfect triplet repeat that meets the minimum 26-repeat threshold of our locus-rich sample, so these can be expected to show the level of diversity shown in Figure 2. Furthermore, there are an additional 1414 coding-sequences with triplet-repeat microsatellites of 11–25 repeats, which will also likely be quite variable.

## Discussion

The extraordinary abundance and variability of amino acid repeats are not easy to explain. If selection is operating, it ought to limit the variation. If it is not, then neutral variation should be a function of the product of population size and mutation rate [29], [30]. The population size of *D. discoideum* is not unusual for a unicellular eukaryote, and the microsatellite slippage rate is unusually low [31]. However *D. discoideum* does have an unusual trait that could be responsible. It has an extremely AT-rich genome – 77.6% overall and 72.5% in coding regions [4], which possibly drives the occurrence of simple sequence repeats, as shown in a previous comparative analysis [32]. Microsatellites often begin as short repeats generated by chance substitutions [9], and these will be much more abundant in a genome that approaches a two-base code than in one where the four bases are used more equally.

The large number and high variability of triplet repeats in *D. discoideum* make it a candidate for the most variable proteome known, but several questions remain unanswered. First, do long amino acid repeats have any function? Microsatellite repeats do sometimes have functional effects, but there would still remain the question of why there is so much diversity. It is unlikely that balancing selection acts on all these loci, as it does on diverse human major histocompatibility complex loci [33] and plant self-incompatibility loci [34]–[36], because of the extremely high cost of selection that would be required over so many loci.

An alternative possibility is that these sequences have little effect on the function of the protein and are not strongly selected. Most of the repeats are of small hydrophilic amino acids [4], which would tend to form loops on the outside of the protein rather than disrupting internal structure. Repeat motifs coding for other amino acids are not highly represented in the genome, presumably because they are deleterious [4]. However, many of the amino acid repeats are glutamine repeats, the same sequences that cause a number of neurodegenerative diseases in humans [13], raising the question of whether *D. discoideum* has some means to protect itself against such deleterious effects. It does not splice the repeat sequences out of the mRNA. The repeats show up in ESTs of the full-length cDNA, and large repeats are not under-represented (Figure S1). It is worth considering whether there might be some novel mechanism for splicing out amino acid repeats from proteins, but some preliminary evidence suggests it does not occur. A western blot (courtesy of William Loomis) of *D. discoideum* protein stained using an antibody that binds to polyglutamines with 30 or more repeats shows a broad smear, suggesting (though not proving) that there really are many proteins bearing such repeats (Figure S2). Future work on the effects of these sequences might be useful for understanding and controlling human glutamine repeat diseases.

The best evidence that these long asparagine repeats are not strongly selected comes from the comparison of paired coding and non-coding loci in Fig. 2. These sets were matched for repeat motif, total length of the repeat sequence, and location in the genome. If repeat regions in coding regions were subject to stronger purifying selection than those in non-coding regions, they should be less variable. They are not less variable, suggesting that purifying selection, if present, is no stronger on the coding repeats.

## Materials and Methods

### Microsatellite Searches

We used the annotated genome of *Dictyostelium discoideum* (http://dictybase.org/) to search for microsatellites located inside coding regions of known genes. We identified microsatellites with perfect repeats, and also accepted long microsatellites with one point mutation in the repeat motif.

### Clones

In the clone-rich sample we genotyped 8 microsatellite loci (Table S1) in 316 clones (Table S2) of *Dictyostelium discoideum* collected from 6 geographic locations: Japan, Massachusetts, North Carolina, Tennessee, Texas, and Virginia. These included 5 asparagine repeats, two glutamine repeats and one lysine repeat. In the multiple-repeat sample, 115 of these clones (Table S2) were genotyped for 8 microsatellite loci from genes *dimA*, *yakA* and *atg1* (Table S1), including five glutamine and three asparagine repeats. In the locus-rich sample we genotyped 50 loci in coding regions (Table S3) for 12 individuals from the USA locations (Table S4) and 12 individuals from a 50 m transect in a Virginia population. To compare variation with that in non-coding regions, we genotyped the same individuals for a sample of non-coding paired for repeat motif, repeat number (average difference 0.80 bp ± s.d. 4.53) and location in the genome (average distance 16.52 kb ± s.d. 14.70). All coding repeats in this sample coded for asparagine repeats.

### DNA Extraction

To obtain genomic DNA, we plated spores of each clone from frozen stocks on SM-agar plates [37] with *Klebsiella aerogenes* bacteria as a food source. Once fruiting bodies developed, which was usually within 3–5 days, we placed 5–10 sori (cluster of spores at the top of the fruiting bodies) in 150 µL of 5% Bio-Rad Chelex-100 and 10 µL of 20 mg/ml proteinase K. We then ran the samples in a PTC-100 programmable thermal controller (step1∶56 C for 4 h; step 2∶98.0 C for 30 min; step 3: End).

### Genotyping

We amplified the microsatellite loci using fluorescently labeled primers (Table S1 and S3) in a polymerase chain reaction (PCR) (step1∶90.0°C for 3 min; step2∶90.0°C for 30 sec; step 3∶60.0°C decreasing 0.5°C every 30 min cycle; step 4∶72.0°C for 30 sec; step 5∶20 cycles to step 2; step 6∶90.0°C for 30 sec; step 7∶50.0°C for 30 sec; step 8∶72.0°C for 30 sec; step 9∶10 times to step 6; step 10∶72.0°C for 10 min; step 8: end). The PCR product was cleaned with ethanol precipitation and then prepared for analysis on an ABI Prism® 3100 Genetic Analyzer. We scored the data using Genotyper software (Applied Biosystems).

### Sequencing

To confirm that differences in allele sizes among clones were due to changes in the number of repeats, and not to changes in the length of the two flanking regions, we sequenced the smallest and the largest alleles of the microsatellites from the 3 genes with known phenotypic effect by amplifying them through PCR with non-fluorescent primers, we then cleaned PCR products with exonuclease I and shrimp alkaline phosphatase (ExoSAP-IT) to remove unincorporated primers and nucleotides, and sent the cleaned product with forward or reverse primers for sequencing (SeqWright DNA Technology Services).

### Data Analysis

We inferred the number of repeats by simply taking off the size of the two flanking regions from the PCR product size and dividing by 3 (i.e., the unit motif). Heterozygosity is the parameter generally used to express the level of polymorphism of a genetic locus. However observed heterozygosity cannot be calculated *for D. discoideum* because it is a haploid organism. Nonetheless we can compute the expected heterozygosity, which is the probability that 2 random alleles in the sample will be different, by considering the frequency of each allele in the population. We did this for all 8 microsatellite loci from genes *dimA*, *yakA* and *atg1* using the Genetic Data Analysis (GDA) software [38]. To obtain a confidence interval on our estimate of expected heterozygosity for each locus, we performed a nonparametric bootstrap using Matlab. To do the bootstrap, we re-sampled with replacement from our original population to have 1,000 new populations and calculated their expected heterozygosity. We used these 1,0000 values to generate a distribution of expected heterozygosity scores at each locus, with the 25th and 975th smallest values delineating the limits of the lower and upper 95% confidence interval, respectively.

## Supporting Information

Figure S1 **Triplet microsatellites in cDNAs.** For each number of repeats ≥5, a blue diamond shows the proportion that are covered, at least in part, in 163,182 *D. discoideum* expressed sequence tags from cDNA (dictyBase 12-19-2008). Pink squares show the fraction of non-repeat sequences in those same genes covered by ESTs. The last point of each color represent is for all repeat numbers greater than 50. At least two possible biases exist, though neither affects the main point that triplet repeats are found in cDNA. First, it is more likely that at least part of a longer repeat will be covered. Second, location of microsatellites in genes may affect representation in ESTs.
(TIF)

Figure S2 **Western blot of** ***D. discoideum*** **proteins stained using an antibody that binds to homopolymer of >30 glutamines.** Each lane shows the total extract of proteins from 0 hour (vegetative stage), 12 and 18 hours (developmental stages), respectively. A 1∶100 dilution of the monoclonal antibody was used. The molecular weight markers are indicated in kDa. Courtesy of Bill Loomis.
(JPEG)

Table S1 **PCR Primer pairs used in the multiple-repeat sample (first eight loci) and the clone-rich sample (last eight).**
(PDF)

Table S2 **Clones genotyped for the clone-rich sample and the multiple-repeat sample.**
(PDF)

Table S3 **PCR primer pairs for the coding loci used in the locus-rich sample.**
(PDF)

Table S4 **Clones in the locus-rich sample.**
(PDF)

Table S5 **All triplet microsatellites with ≥5 repeats in genes** ***yakA*** **,** ***dimA*** **and** ***atg1*** **from the reference genome of** ***D. discoideum*** **AX4, including the 8 genotyped in the multiple-repeat sample (boldface).**
(PDF)
