# Supplementary figures and images for: Amino Acid Repeats Cause Extraordinary Coding Sequence Variation in the Social Amoeba Dictyostelium discoideum
Source: PLoS One. 2012 Sep 28;7(9):e46150. doi: 10.1371/journal.pone.0046150 (PMC3460934; doi:10.1371/journal.pone.0046150)

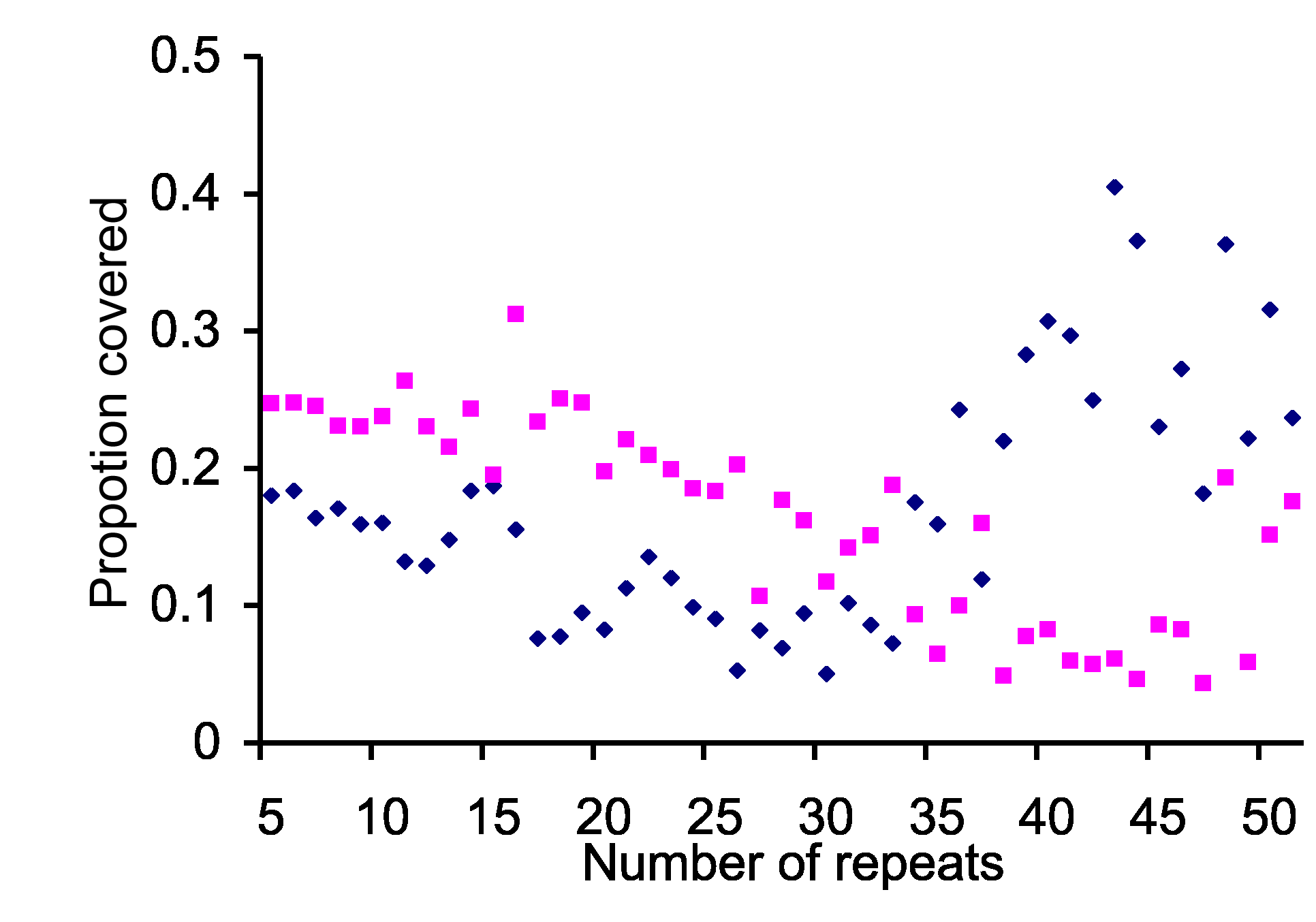

Supplement: Figure S1 — Triplet microsatellites in cDNAs. For each number of repeats ≥5, a blue diamond shows the proportion that are covered, at least in part, in 163,182 D. discoideum expressed sequence tags from cDNA (dictyBase 12-19-2008). Pink squares show the fraction of non-repeat sequences in those same genes covered by ESTs. The last point of each color represent is for all repeat numbers greater than 50. At least two possible biases exist, though neither affects the main point that triplet repeats are found in cDNA. First, it is more likely that at least part of a longer repeat will be covered. Second, location of microsatellites in genes may affect representation in ESTs. (TIF) [file pone.0046150.s001.tif]

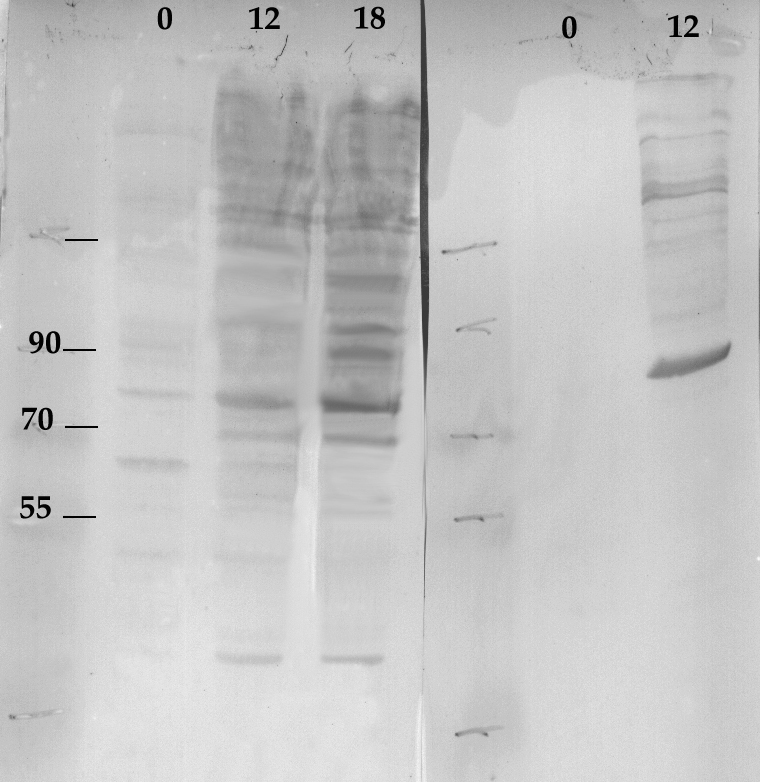

Supplement: Figure S2 — Western blot of D. discoideum proteins stained using an antibody that binds to homopolymer of >30 glutamines. Each lane shows the total extract of proteins from 0 hour (vegetative stage), 12 and 18 hours (developmental stages), respectively. A 1∶100 dilution of the monoclonal antibody was used. The molecular weight markers are indicated in kDa. Courtesy of Bill Loomis. (JPEG) [file pone.0046150.s002.jpeg]
