# Supplementary material for: Amino Acid Repeats Cause Extraordinary Coding Sequence Variation in the Social Amoeba Dictyostelium discoideum
Source: PLoS One. 2012 Sep 28;7(9):e46150. doi: 10.1371/journal.pone.0046150 (PMC3460934; doi:10.1371/journal.pone.0046150)
Supplement: Table S1 — PCR Primer pairs used in the multiple-repeat sample (first eight loci) and the clone-rich sample (last eight). (PDF) [file pone.0046150.s003.pdf]

Table S1. PCR Primer pairs used in the multiple-repeat sample (first eight loci) and the clone-rich sample (last eight).

Gene	Access number	Locus	Motif	Primer pair
dimA	DDB0216189	dimA1	AAT	5'-CCAACCATTTTCACTTCAACC-3'<br>5'-GTCGATTATTAAATGTTTGCGGTT-3'
		dimA2	CAA	5'-GGTAATGATAACACTTTACCACCTT-3'<br>5'-GTCGTTGTTGGTGTTGTTGT-3'
		dimA3	AAT	5'-CACCACGTCCACCATTAGAT-3'<br>5'-TTACCGTTATTATTGTTGCCATT-3'
yakA	DDB0191191	yakA1	CAA	5'-TCAACACCAACTTTTTCAACAAT-3'<br>5'-AGTAGTAGTGTTGTTGTATATGTCT-3'
		yakA2	CAA	5'-CAGAGGGAATGGATCCACAA-3'<br>5'-AACGTCATGGTTGTTGTTAAGG-3'
		yakA3	CAA	5'-GACCCAGAGGTGATTCTATGAAA-3'<br>5'-GGAAGAGAGTACTACAGTGGT-3'
ATG1	DDB0185178	ATG1-1	AAT	5'-ATTCATATAAAAATAATAGCGGAAA-3'<br>5'-CAAATGGTAAACGTATGTTATTGTT-3'
		ATG1-2	CAA	5'-AACCGAGGCAATACCACAAC-3'<br>5'-GTCTGTTTTGCTTGGTAACC-3'
BC4V2_0_01554	DDB0218783	Locus 5	CAT	5'-GATAGAGAAACTGACACTTGGG-3'<br>5'-GGTGGAGCTTTATTGTCTACC-3'
JC1V2_0_00837	DDB0190016	Locus 19	AAC	5'-GCTTGATTTGCCAATAGTTC-3'<br>5'-TCAAAACCTGATCCATTACC-3'
JC1V2_0_00905	DDB0202354	Locus 25	AAC	5'-AGAGCCACTCATTATCTATTCC-3'<br>5'-CACAACCACTATCACTAGAAACTG-3'
JC1V2_0_00551	DDB0189741	Locus 431	AAC	5'-CCACAACTTCAACAAACCC-3'<br>5'-CAACACTTTATCGGATGATTG-3'
BEC6V2_0_00596	DDB0184365	Locus 200	CAT	5'-CACTTGCTTCTTCTGCTTCATATTC-3'<br>5'-TTCACTGATGGTGGTGTTATACC-3'
JC3V2_0_01755	DDB0218298	Locus 715	AAC	5'-GGTTCACATAATCGTTTTGCTTTGG-3'<br>5'-TTCACTGATGGTGGTGTTATACC-3'
BC5V2_0_01494	DDB0219539	Locus 20	AAC	5'-TTGGGCATTATCATCCTC-3'<br>5'-CTTCTTTGTTGGTGTCATTG-3'
elmoE	DDB0233920	Locus 325	AAG	5'-TTCCACCATCTTTATCTCCAAC-3'<br>5'-CACTCTTTGTATCATCATCGTCATC-3'
