# Supplementary material for: Amino Acid Repeats Cause Extraordinary Coding Sequence Variation in the Social Amoeba Dictyostelium discoideum
Source: PLoS One. 2012 Sep 28;7(9):e46150. doi: 10.1371/journal.pone.0046150 (PMC3460934; doi:10.1371/journal.pone.0046150)
Supplement: Table S2 — Clones genotyped for the clone-rich sample and the multiple-repeat sample. (PDF) [file pone.0046150.s004.pdf]

**Table S2. Clones genotyped for the clone-rich sample and the multiple-repeat sample**

| Location | | Number of clones* | |
|---|---|---|---|
| | Massachusetts (MA) | 34 | (18) |
| | North Carolina (NC) | 31 | (20) |
| USA | Tennessee (TN) | 53 | (14) |
| | Texas (TX) | 47 | (16) |
| | Virginia (VA) | 147 | (47) |
| Japan | | 4 | (0) |

*The number of clones is shown for clone-rich sample; the subset used in the multiple-repeat sample is shown in parentheses.
