# Supplementary material for: Amino Acid Repeats Cause Extraordinary Coding Sequence Variation in the Social Amoeba Dictyostelium discoideum
Source: PLoS One. 2012 Sep 28;7(9):e46150. doi: 10.1371/journal.pone.0046150 (PMC3460934; doi:10.1371/journal.pone.0046150)
Supplement: Table S3 — PCR primer pairs for the coding loci used in the locus-rich sample. (PDF) [file pone.0046150.s005.pdf]

Table S3. PCR primer pairs for the coding loci used in the locus-rich sample.

Chromosome	Access number	Locus	Motif	# of Repeats	Primer Pairs
1	DDB0190057	398A	AAT	31	5'-CAAAAATATCATCAATTTCAATTCCA-3'<br>5'-TGGAGTGATTCAATTATTGGTAAA -3'
1	DDB0190902	395A	AAT	34	5'-CAGAACCTGCATTTGGAGCTA-3'<br>5'-CATCATCACCATTCCCATCA-3'
1	DDB0190549	399A2	AAT	37	5'-TTCATCTTCAACAACAGGCAGT- 3'<br>5'-GGTTGAGTTTGTGCTTGTGG-3'
1	DDB0202354	400A	AAC	27	5'-AACATTGGCAAAAAGAGTTGC-3'<br>5'-TTCACCAAGTCCTACTTCTCCAA-3'
1	DDB0189545	401A	AAT	32	5'-GGTGTTGTTGTTGGTGTTGG-3'<br>5'-TCCAATTTCAAATCCATTTTGT-3'
1	DDB0201836	402A'	AAT	28	5'-TGACGATGGTGAAGAAGAAGG-3'<br>5'-GGTGATAATGGCACCTCAACA-3'
1	DDB0216601	403A'	AAT	29	5'-GGTGTTGTTGTTGGTGTTGG-3'<br>5'-TCCAATTTCAAATCCATTTTGT-3'
1	DDB0189622	404A2	AAT	30	5'-TGACGGGTGACGGATTTATT- 3'<br>5'-AAAAATGAAAAACTATCACACGAA-3'
2	DDB0217236	200E	AAT	28	5'-CAGATTGAACAGTATTCGCAGA-3'<br>5'-ACCATTACCTTCCCCATTCC-3'
2	DDB0217267	201E	AAT	30	5'-CCAATAAAAATGTTGCAACTGA-3'<br>5'-TGATTTCTCTCCACCTTTCCA-3'
2	DDB0216955	501A	AAT	28	5'-TGTGGTATCAAATCACCAATTCA-3'<br>5'-AAATTTGTTGTGTTTCATCTAATGTT-3'
2	DDB0167992	503E	AAT	32	5'-TGCACCATGTTTTTGAATATGA-3'<br>5'-GGTTGAAGGTAATCATATTGTTGG-3'
2	DDB0217129	504A	AAT	31	5'-TGATTTCTCTCCACCTTTCCA-3'<br>5'-CCAATAAAAATGTTGCAACTGA-3'
2	DDB0217194	505E	AAT	28	5'-TTCATCAACAACAATCTCAACAA-3'<br>5'-TTGGTGGTGATGTTTCAGGT-3'
2	DDB0167853	506E	AAT	34	5'- AAACCAGCCAATACTGGATCA-3'<br>5'-CCAACACCCATTACAGCAAC-3'
2	DDB0169026	509E	AAT	27	5'-TCTGGTGAATTAAATATTTTTCTTCTT-3'<br>5'-CCACATAATGGTTTTCCTGTTG-3'
2	DDB0168144	510E	AAT	28	5'-ACCATTACCTTCCCCATTCC-3'<br>5'-CAGATTGAACAGTATTCGCAGA-3'
3	DDB0204301	511E	AAT	29	5'-TCCACTTATCGTGGTTTTTGG-3'<br>5'-TATCTCCACCATTGCAACCA-3'
3	DDB0205758	513E2	AAT	30	5'-CCTGTTGCACCAGAGGCTAT-3'<br>5'-TTGGTTATTGTTTGGCTGGTT-3'
3	DDB0204352	514E	AAT	33	5'-ATACCGAGCATATGGTGCAA-3'

					5'-AACCTCAATGGGACCATCAC-3'
3	DDB0206405	515E	AAT	29	5'-ACAAATAAAGGAGGAGTACCATTG-3'<br>5'-TCCAATTTCTGGATGTTCACC-3'
3	DDB0203942	516E	AAT	40	5'-AATCGTACCCATTGGCATCA-3'<br>5'-CCATCACCATCACCACAAAA-3'
3	DDB0205183	517E	AAT	28	5'-AAATGTGAAATCATAAATTGAGGTAA-3'<br>5'-TTGTTCAAAGAATTAATAATGAAACC-3'
3	DDB0204159	518E	AAT	30	5'-GAAATGCTCATTACTTGGAAAAA-3'<br>5'-TGCTTGTGATGCTAAAATTGA-3'
3	DDB0204611	519E	AAT	32	5'-TGTTAAAGGATTTTCATCCTCTTCT-3'<br>5'-TGTACCAAATAACCCACCATCA-3'
3	DDB0205026	521E	AAT	27	5'-CGCCTCAAACATTCCCAGTA-3'<br>5'-CCTCCATTTACTGTTTGTGATTTT-3'
4	DDB0218521	600A	AAT	30	5'-TGGTGATCCTGAAATTTGATCT-3'<br>5'-CACCAATGGTACCAACTCCA-3'
4	DDB0185366	603A	AAT	27	5'-TTTCCAATATTTCCATTATTACCC-3'<br>5'-AATTCAAGCAATCCATCATGC-3'
4	DDB0185789	604A	AAT	27	5'-TGAATTTTTGGTTCCAACTTGT-3'<br>5'-TCATTTTATGGTAGTGAAAAAGCA-3'
4	DDB0186356	606E	AAT	29	5'-AAAATTTGATGTGGTAATGAAATAAGT-3'<br>5'-AAAACCTACAAATATTGATTCAGAAAA-3'
4	DDB0186443	607E	AAT	37	5'-CCAATCTCCAATCATACACTCG-3'<br>5'-TCGGCACTAGTTGAAATTCG-3'
4	DDB0218811	608E	AAT	28	5'-AAACGGGTGGTAGGAATGGT-3'<br>5'-AAGTTGTTGTTTAATTGCGAATG-3'
4	DDB0187170	609E	AAT	32	5'-TGCCAATTAATGGAATGACTTTT-3'<br>5'-TCAATAGATGTTGAAAGATCATGGTT-3'
4	DDB0186229	612E	AAT	30	5'-TCTTGATTTTCCATAATTTTTATTGGT-3'<br>5'-TGATGAAGAGCTTTTGGAGGA-3'
4	DDB0347517	612E	AAT	30	5'-TCTTGATTTTCCATAATTTTTATTGGT-3'<br>5'-TGATGAAGAGCTTTTGGAGGA-3'
5	DDB0187463	405A'	AAT	30	5'-TTTTTAGAATCAATGGGTGGA-3'<br>5'-TGATTCCAAGGGTTCATTTG-3'
5	DDB0219263	406A	AAT	37	5'-AAAATCTTGTTGTTCAGTCGTAGG-3'<br>5'-TTGTTTATTAATTTGCATCGATTT-3'

5	DDB0219326	407A	AAT	31	5'-TGAGTTATAACCTGAACAGAAGATGG-3'<br>5'-TTTCAACCAACATTTTTAAAGACAA-3'
5	DDB0188232	408A	AAT	30	5'-CAATCTTATGGCGATGATGAAA-3'<br>5'-TGAAGTTGGAGAGATGGATGG-3'
5	DDB0219647	409A	AAT	39	5'-TGCTAATCTTTCACCTTGTCCA-3'<br>5'-CCTCAAACTTATGCCAACAACA-3'
5	DDB0187587	412A	AAT	29	5'-TCAATTGGTATTGAGGATGCAA-3'<br>5'-TTTTTGATCTTCTTTAATTGCTTTCA-3'
5	DDB0346576	413A2	AAT	40	5'-CAACATCAACAACAACATCAACA-3'<br>5'-CAACTGATAATAATGAGTCACCAAA-3'
5	DDB0188704	414A'	AAT	28	5'-TCAAACCATTAAATTCGATAACCA-3'<br>5'-CCACAACCTTCTCCAAATGC-3'
6	DDB0348248	415A'	AAT	28	5' -TTGGCAATTAGCTGGTGAAA-3'<br>5'-TTCAATTCCATCATCTTTCCAA-3'
6	DDB0183813	416A	AAT	28	5'-TCAAAACGGTCAAGTAATTCCA-3'<br>5'-AAAGGAAAACTCCAAAATTGATG-3'
6	DDB0184314	417A	AAT	34	5'-TTTGTTGAGTTTCCTCTGCACT-3'<br>5'-CAACGACCAAGATAATATAAGCGATA-3'
6	DDB0191672	418A2	AAT	30	5'-TGGTGGTGGTGGTGTTAATG-3'<br>5'-TGTGGACTAAAAATCATTGGACT-3'
6	DDB0219853	419A	AAT	32	5'-GCTTCAACTTCCACAACCATC-3'<br>5'-TGCTCCTTTAATTGGATACCATC-3'
6	DDB0238049	423A'	AAT	29	5'-TTCTTCTTCCTCTACTTCCTCTTCT-3'<br>5'-AAGTGTTGGAATGTTTCCCATT-3'
