# Supplementary material for: Amino Acid Repeats Cause Extraordinary Coding Sequence Variation in the Social Amoeba Dictyostelium discoideum
Source: PLoS One. 2012 Sep 28;7(9):e46150. doi: 10.1371/journal.pone.0046150 (PMC3460934; doi:10.1371/journal.pone.0046150)
Supplement: Table S4 — Clones in the locus-rich sample. (PDF) [file pone.0046150.s006.pdf]

Table S4. Clones in the locus-rich sample.

	Clones	Location
USA clones	HD37	Texas - Houston Arboretum
	MA12	Massachusetts - Mt. Greylock
	NC28	North Carolina - Little Butts Gap
	S109	Arkansas - Forest City
	S202	Texas - Carthage
	S203	Illinois - Effingham
	S221	Missouri - St. Louis
	S30	Virginia - Mountain Lake Biological Station
	S67	Texas - Linden
	S71	Indiana - Bloomington (Lobelia)
	S72	Kentucky - Land Between the Lakes
	TN39	Tennessee - Indian Gap
Virginia clones	V301B1	Virginia - Mountain Lake Biological Station
	V303A2B	Virginia - Mountain Lake Biological Station
	V305B2	Virginia - Mountain Lake Biological Station
	V316A1	Virginia - Mountain Lake Biological Station
	V319A	Virginia - Mountain Lake Biological Station
	V323D1	Virginia - Mountain Lake Biological Station
	V326D1	Virginia - Mountain Lake Biological Station
	V330B1	Virginia - Mountain Lake Biological Station
	V331C2	Virginia - Mountain Lake Biological Station
	V335B1	Virginia - Mountain Lake Biological Station
	V337D1	Virginia - Mountain Lake Biological Station
	V342A2	Virginia - Mountain Lake Biological Station
