# Supplementary material for: Amino Acid Repeats Cause Extraordinary Coding Sequence Variation in the Social Amoeba Dictyostelium discoideum
Source: PLoS One. 2012 Sep 28;7(9):e46150. doi: 10.1371/journal.pone.0046150 (PMC3460934; doi:10.1371/journal.pone.0046150)
Supplement: Table S5 — All triplet microsatellites with ≥5 repeats in genes yakA , dimA and atg1 from the reference genome of D. discoideum AX4, including the 8 genotyped in the multiple-repeat sample (boldface). (PDF) [file pone.0046150.s007.pdf]

Table S5. All triplet microsatellites with ≥ 5 repeats in genes *yakA*, *dimA* and *atg1* from the reference genome of *D. discoideum* AX4, including the 8 genotyped in the multiple-repeat sample (boldface).

Gene	Gene Length	MS ID *	MS Motif	MS Repeats	MS Start	MS End
yakA	4377	**1**	**CAA**	**54**	**1780**	**1941**
		**2**	**CAA**	**32**	**2641**	**2736**
		**3**	**CAA**	**31**	**4042**	**4134**
		4	AAC	13	157	195
		5	TAA	13	2454	2492
		6	ATA	9	1349	1375
		7	CAA	7	3217	3237
		8	TAA	6	210	227
		9	TAA	6	2535	2552
		10	ACA	6	3597	3614
		11	ACA	5	2184	2198
		12	CAA	5	3100	3114
dimA	3693	**1**	**CAA**	**63**	**625**	**813**
		**2**	**AAT**	**45**	**3154**	**3288**
		**3**	**AAT**	**31**	**223**	**315**
		4	TCA	9	1474	1497
		5	CAT	8	2497	2520
		6	TCA	7	1569	1589
		7	ACA	6	1128	1145
		8	ACA	6	1456	1473
atg1	2007	**1**	**TAA**	**35**	**939**	**1043**
		**2**	**CAA**	**27**	**1330**	**1410**
		3	TAA	14	861	902
